# The Diverse Roles of Auxin in Regulating Leaf Development

**DOI:** 10.3390/plants8070243

**Published:** 2019-07-23

**Authors:** Yuanyuan Xiong, Yuling Jiao

**Affiliations:** 1 State Key Laboratory of Plant Genomics and National Center for Plant Gene Research (Beijing), Institute of Genetics and Developmental Biology, The Innovative Academy of Seed Design, Chinese Academy of Sciences, Beijing 100101, China; 2 College of Life Sciences, University of Chinese Academy of Sciences, Beijing 100049, China

**Keywords:** auxin, leaf blade, auxin transport, patterning, auxin signaling, compound leaf

## Abstract

Leaves, the primary plant organs that function in photosynthesis and respiration, have highly organized, flat structures that vary within and among species. In recent years, it has become evident that auxin plays central roles in leaf development, including leaf initiation, blade formation, and compound leaf patterning. In this review, we discuss how auxin maxima form to define leaf primordium formation. We summarize recent progress in understanding of how spatial auxin signaling promotes leaf blade formation. Finally, we discuss how spatial auxin transport and signaling regulate the patterning of compound leaves and leaf serration.

## 1. Introduction

Unlike animals, plants continuously produce new organs, forming leaves, flowers, and stems postembryonically. The leaf is a determinate lateral organ. Leaves bulge out at the flanks of the indeterminate shoot apical meristem (SAM), a highly organized tissue containing stem cells. A typical eudicot leaf is composed of two morphologically distinct parts: A broad, flat blade and a narrow, stem-like petiole. For most plant species, leaves are the major sites of photosynthesis. The upper side of the blade is usually specialized for light capture and the lower side for gas exchange. The presence of broad, flat blades not only maximizes photosynthesis, but it also increases water loss. Thus, blade shape is often a critical trait for the environmental adaption and agronomic value of a plant.

**Figure 1:**
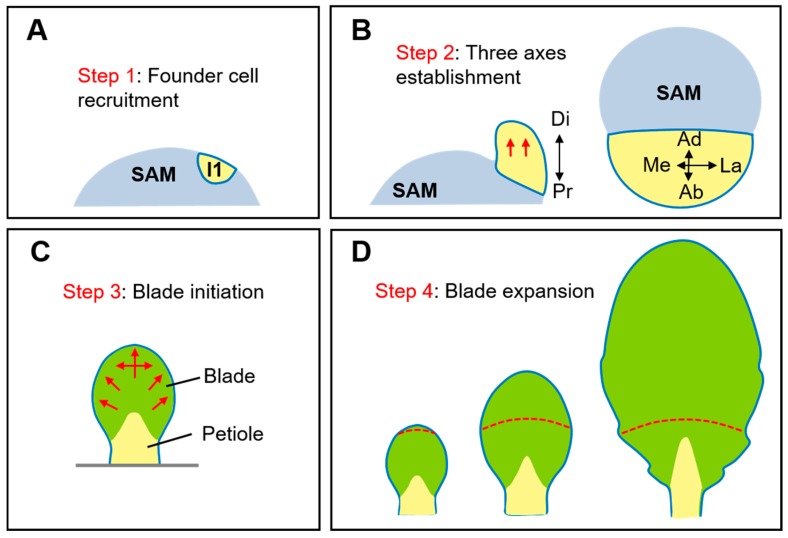
Schematic illustration of four steps during leaf development. (**A**) The founder cells of leaf primordium are recruited from the flank of the SAM. I1, the oldest incipient primordium. (**B**) After initiation, the leaf primordium grows predominantly in the distal direction and establishes three growth axes: adaxial-abaxial axis (Ad-Ab), proximal-distal axis (Pr-Di), and medio-lateral axis (Me-La). Left, front view. Right, top view. (**C**) Afterwards, the blade initiates from the marginal regions and grows along medial-lateral axis to separate blade from petiole. (**D**) Finally, the blade undergoes rapid expansion, which is accompanied by cell division and cell differentiation occurring in the entire blade. The transition from cell division to differentiation shift basipetally in *Arabidopsis* but can be divergent in other species. Red arrows in (**A**–**C**) indicate the direction of leaf growth. Red lines in (**D**) indicate the arrest front. Modified from [1,2].

Whereas leaf size and shape vary within and between species, these traits share similar developmental pathways. Leaf development involves four continuous, overlapping processes [1,2] (Figure 1). First, the founder cells of the leaf primordium are recruited from the peripheral zone (PZ) of the SAM and bulge out. Second, an initiated leaf primordium develops in three principal growth axes, including the adaxial-abaxial (also called dorsoventral) axis in the up-down direction, the medio-lateral (centrolateral) axis in the middle-to-side direction, and the proximal-distal (proximodistal) axis in the longitudinal direction of the leaf. Third, the leaf blade initiates from the marginal region between the adaxial and abaxial sides and expands to give rise to the basic leaf form. Finally, the blade undergoes more rapid expansion than the petiole to reach the final leaf shape, in which cell division and cell differentiation participate. The transition from cell division to cell differentiation occurs in a basipetal manner in many species, including the model plant *Arabidopsis* (*Arabidopsis thaliana*) (Figure 1D). In such species, the forefront of cycling cells, named the “arrest front”, moves basipetally [3,4]. In other species, the arrest front can move acropetally, such as in tobacco (*Nicotiana tabacum*) [5], bidirectionally, or diffusively without clear gradient [6].

Leaf shape is determined by internal cues and is influenced by external factors, such as water, light, temperature, and pathogens. Like many morphogenetic processes, each step of leaf development can be roughly divided into patterning and shaping. During leaf patterning, the differential expression of gene products and differential distribution of phytohormones are established, which direct differential cell division and growth to elicit shape changes. The classic phytohormone, auxin, is involved in each step of leaf development and often bridges leaf patterning with shaping. Here, we discuss how auxin regulates leaf initiation, polarity, flattening, and shape diversity.

## 2. The Role of Auxin in Regulating Leaf Initiation

Studies over the past two decades have shown that auxin plays a crucial role in leaf initiation. Tomato (*Solanum lycopersicum*) vegetative shoot apices treated with the auxin transport inhibitor 1-*N*-naphthylphthalamic acid (NPA) fail to form leaf primordia, whereas the meristem continues to generate stem tissue, leading to the formation of a pin-like structure lacking leaves. Similarly, organ formation is blocked in the inflorescence meristems of *pin-formed1* (*pin1*) mutants in *Arabidopsis*, which harbor mutations in the auxin efflux carrier PIN1 [7,8,9]. Exogenous auxin application restores organ formation in both NPA-treated tomato shoot apices and *Arabidopsis pin1-1* inflorescence apices [10]. Accordingly, the formation of auxin response maxima (as revealed using the synthetic auxin-induced transcriptional reporter DR5) precedes leaf primordium initiation [11,12].

The formation of auxin response maxima is thought to be related to PIN1 convergence points in the epidermis [11,13,14]. Epidermal PIN1 in the SAM is polarized toward cells with high auxin concentrations that are destined to form an incipient primordium. PIN1 convergence as suggested by computational modeling, might explain the regular arrangement of lateral organs around the stem, termed phyllotaxis. As in many species, the model plants *Arabidopsis* and tomato have spiral phyllotaxy, in which a new primordium emerges ~137.5° from the previous one. Based on the assumption that PIN1 is arranged toward neighboring cells with higher auxin concentrations, computational models have been developed that recapitulate the convergence of directional auxin transport toward incipient primordia. Each auxin maximum is accompanied by auxin depletion at its periphery, which blocks organ initiation in this region [15]. These models predict the formation of periodic, self-organized auxin maxima, which recapitulate phyllotaxy [16,17,18]. A key assumption is that positive feedback occurs between auxin and PIN1 localization, with PIN1 transporting auxin towards cells with higher auxin levels. It has been proposed that cells compare their own auxin concentrations with those of neighboring cells and orient PIN upwards auxin gradients [16,17]. Alternatively, PIN1 might arrange itself in a polar manner towards expanding neighboring cells with higher auxin levels [18]. Both of these theories require further experimental validation.

However, this viewpoint has been challenged by recent studies involving high-resolution imaging of the localization of PIN1 and the auxin biosensor DII-Venus. According to these studies, the central zone of the SAM generally contains high levels of auxin, although low levels of auxin signaling occur in this region [19,20]. The central high-auxin zone forms periodical protrusions that coincide with incipient primordia, which is specified by the duration of cell exposure to auxin. The duration of auxin exposition may explain the robust and rhythmical phyllotactic patterning [20]. Further, and contrary to many prior published results, auxin flow in incipient and newly-formed primordia largely points toward the SAM, thus creating the previously observed high auxin zone. This is in contrast to the previously assumed “up the gradient” in incipient and emerging primordia. While PIN polarities converge toward the SAM center, they also form local regions of convergence close to the auxin maxima and protrusions [20,21]. Convergence of PIN1 at each primordium leads to a low-auxin zone at the adaxial side of each primordium. Part of the low-auxin zone encompasses the boundary, which separates the primordium from the SAM. The NAC family genes, such as *Arabidopsis CUP-SHAPED COTYLEDON* (*CUC*) genes, are expressed in the organ boundary, and are repressed by auxin [9,22,23]. The low-auxin zone also extends into the primordium and contributes to leaf polarity patterning. On the other hand, auxin flow out of two neighboring adaxial low-auxin zones toward the SAM, which may lead to the formation of an incipient primordium around the peripheral zone of the SAM on the opposite side. Notably, these observations were primarily obtained using inflorescences; it is crucial to perform high-resolution imaging of the vegetative SAM as well. Nevertheless, it has been found in the tomato vegetative shoot apex that PIN1 convergence also points toward leaf margins, where leaf blade forms [21].

In addition to the auxin efflux carrier PIN1, auxin influx carriers encoded by *AUXIN1*/*LIKE-AUX1* (*AUX1*/*LAX*) family genes stabilize phyllotaxy [24]. Auxin directly activates the expression of the cytokinin signaling inhibitor ARABIDOPSIS HISTIDINE PHOSPHOTRANSFER PROTEIN6 (AHP6), which moves intercellularly to generate inhibitory fields of cytokinin signaling and contributes to the robustness of phyllotaxis [25]. Auxin biosynthesis mediated by YUCCA (YUC) flavin monooxygenases also helps determine phyllotaxy. In the *Arabidopsis* shoot apex, *YUC4* is expressed in the abaxial side of the primordium, which might be important for maintaining the robustness of phyllotaxy [20]. Furthermore, introducing *pin1* into the *yuc1 yuc4* double mutant or *aux1* into the *yuc1 yuc2 yuc4 yuc6* quadruple mutant background completely suppressed postembryonic leaf initiation [26]. Transcription factors such as PLETHORA proteins control the expression of *YUC1* and *YUC4* to stabilize phyllotaxis [27].

How auxin signaling maxima are translated into leaf initiation is not yet fully understood. AUXIN RESPONSE FACTOR (ARF) proteins function as transcriptional activators downstream of auxin signaling. ARF activators are released by auxin to transcribe auxin response genes [28]. Repressor ARF competes with activator ARF to inhibit downstream gene expression. The transcription factor ETTIN is responsive to changes in auxin levels [29], but whether other repressor ARF suppressors are also auxin responsive remains to be determined. Two ARF activators, ARF5 (also known as MONOPTEROS [MP]) and ARF7 (also known as NONPHOTOTROPIC HYPOCOTYL4 [NPH4]) redundantly promote leaf primordium initiation, which is significantly compromised in the *mp nph4* double mutant [30,31]. Note that *pin1* mutants and higher-order *pin* mutants only exhibit delayed leaf initiation [32]. By contrast, floral meristem initiation is more strongly affected in *pin1*, leading to the pin-like inflorescence phenotype. However, *mp pin1* double mutants show a leafless phenotype [33], pointing to the presence of a PIN-independent auxin transport mechanism. The tomato *leafless* (*lfs*) mutant does not form leaves [34]. *LFS* is the tomato ortholog of *Arabidopsis DORNRÖSCHEN* (*DRN*) and *DRN*-*LIKE* (*DRNL*), encoding AP2 family transcription factors [34]. Auxin signaling rapidly induces *LFS* expression, although it is likely regulated by other factors in addition to auxin [34].

Class I KNOTTED1-LIKE HOMEOBOX (KNOX1) transcription factors are specifically expressed in the SAM, whereas ASYMMETRIC LEAVES1/ROUGH SHEATH2/PHANTASTICA (ARP) MYB-domain transcription factors are specifically expressed in leaf primordia [35,36,37,38,39]. *KNOX1* genes are specifically expressed in the SAM to maintain its activity, whereas *ARP* genes are specifically expressed in initiating and developing leaf primordia to help determine leaf fate. Auxin interacts with KNOX1/ARP, although the detailed molecular mechanism remains elusive [14,40].

## 3. The Role of Auxin in Regulating Leaf Shape

After initiation, the leaf primordium generally assumes and maintains a flattened structure. As mentioned above, leaves are three-dimensional organs whose growth along the adaxial-abaxial, medio-lateral, and proximal-distal axes is not uniform. As a result, leaf primordia become asymmetric and flattened along the medio-lateral axis. The formation of the proximal-distal axis accompanies leaf initiation, as does the formation of the adaxial-abaxial axis. It has long been thought that a prepatterning of adaxial and abaxial genes occurs around the SAM and that a leaf primordium encompasses the two regions [41]. The existence of this prepatterning has been supported by live and time-lapse imaging of the expression of the adaxial-promoting gene *REVOLUTA* (*REV*) and the abaxial-promoting gene *KANADI1* (*KAN1*) [42,43]. These two genes are expressed in a concentric pattern, with the adaxially expressed *REV* in the center, regardless of whether a leaf primordium is initiated. A complex gene regulatory network involving transcription factors and small RNAs functions downstream of the prepatterned HD-ZIPIII and KAN proteins. In short, this regulatory network involves the mutual repression of adaxial-promoting and abaxial-promoting genes. For a more detailed description of this concept, we refer the reader to recent reviews on adaxial-abaxial polarity [2,44,45,46,47]. The relationship between auxin and the prepatterned polarity genes remains to be fully resolved. Misaccumulation of PIN1 gradients was found in the hypocotyles of *kan1 kan2 kan4* triple mutant embryos [48]. In addition, prolonged co-treatment of auxin perception antagonist auxinole, and auxin biosynthesis inhibitors yucasin and kyn led to expansion and restriction of KAN1 and REV expression toward the SAM center, respectively [43]. It remains to be tested whether auxin directly regulates the expression of *REV* and/or *KAN1*, or auxin affects the SAM homeostasis, as recently suggested [19], and indirectly affects the prepattern.

During leaf initiation, a third domain is established between the adaxial and abaxial domains [42,43]. The middle domain is marked by the expression of two homologous *WUSCHEL-RELATED HOMEOBOX* (*WOX*) genes, *WOX1* and *PRESSED FLOWER* (*PRS*)/*WOX3* [49,50]. *WOX1* and *PRS* redundantly promote cell proliferation to establish the leaf margin and to induce blade outgrowth and flattening [49,50,51]. Indeed, *wox1 prs* double mutants have defects in leaf blade formation, which is highly conserved among a wide range of eudicots and monocots [49,52,53,54,55,56,57].

It has long been thought that adaxial-abaxial polarity conditions the further establishment of medio-lateral polarity and subsequent leaf flattening [51,58], and a recent study showed that auxin is involved in this pattern translation [51]. During early leaf development, a low-auxin domain is established in the boundary region, which separates the primordium from the SAM and extends into the adaxial domain [59,60,61]. Similarly, auxin levels are also low in early floral primordia [20]. Imaging and genetic data support a model in which auxin and prepatterned genes cooperate to establish *WOX* expression and leaf flattening. Spatial auxin signaling transforms adaxial-abaxial polarity into the middle domain to mediate leaf flattening. The ARF activator MP is specifically expressed in the adaxial domain [51,61,62]. However, low levels of auxin in the adaxial domain prevent the ectopic activation of MP and restrict auxin signaling to the middle domain, as observed using the DR5 reporter [51]. In particular, MP, when activated by auxin, directly activates *WOX1* and *PRS* expression [51].

**Figure 2:**
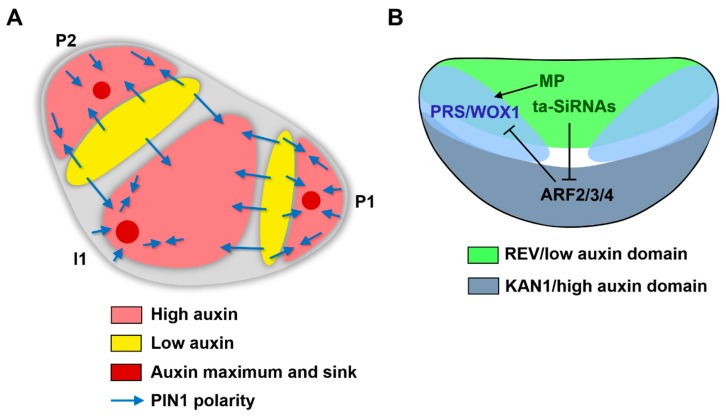
Models of the regulation of leaf initiation and early patterning by auxin. (**A**) Model of the regulation of leaf patterning by auxin polar transport in the epidermis and spatial auxin distribution. Note that auxin maxima are also auxin sinks, where PIN1 directs auxin transport into inner cells. Modified from [20,21]; (**B**) Model of the regulation of leaf patterning by spatial auxin distribution and signaling. Modified from [51].

In the leaves of *pMP::MPΔ* plants (in which MP is active in both the absence and presence of auxin), *WOX1* and *PRS* expression extends into the adaxial domain, where blade-like extrusions form ectopically [51,61,63]. In the abaxial domain, auxin signaling is inhibited by the redundant ARF suppressors ETT, ARF2, and ARF4, whose expression is restricted by adaxially expressed *trans*-acting small interfering RNAs (ta-siRNAs) [64,65,66]. Like MP, these ARF suppressors bind to the promoters of *WOX1* and *PRS*, but they suppress their expression [51]. Moreover, *KAN* genes also repress the expression of *WOX1* and *PRS*. *KAN1* and *KAN2* repress the expression of *WOX1* and *PRS* in the abaxial domain, which is supported by the misexpression of *WOX1* and *PRS* in the *kan1 kan2* double mutant [49]. Because KAN proteins can physically interact with the ETT protein [67], these two types of proteins may form heterodimers to inhibit *WOX1* and *PRS* expression. The expression of adaxial-expressed ARF activators and abaxial-expressed ARF suppressors might be determined by prepatterned adaxial-abaxial polarity. Therefore, auxin distribution and signaling help prepatterned adaxial-abaxial polarity to be translated into medio-lateral polarity and blade formation (Figure 2). On the other hand, *WOX1* and *PRS* (and orthologs in other species) regulate auxin levels and auxin response to promote leaf development [53]. A genome-wide analysis found that both upregulated and downregulated genes by *WOX1* were enriched with auxin responsive genes [68].

The timing of the establishment of the adaxial low-auxin domain is a matter of debate [69,70]. Bhatia et al. argued that the *35S* promoter, when used to drive the expression of the DII-Venus sensor, showed enhanced activity in the adaxial regions of very early leaf primordia [69]. However, Guan et al. argued that the imaging data obtained in that study [69] were biased due to the shape of the tissue, which could have distorted the signals when whole-mount imaging was performed, and that the analysis was artificially biased due to arbitrary domain demarcation [70]. To explain how auxin-responsive *WOX* expression is excluded from the adaxial domain, Bhatia et al. reasoned that HD-ZIPIII transcription factors such as REV restrict auxin responses and *WOX1* and *PRS* expression [69]. Unfortunately, this hypothesis contradicts the experimental finding that the expression patterns of *PRS* and *REV* significantly overlap [43,51,70].

The domain-specific distribution of auxin relies on auxin transport. As mentioned above, recent high-resolution imaging of PIN1 localization indicated that auxin transport occurs from the outside to the center of the SAM in tomato and *Arabidopsis* [20,21]. After leaf emergence, both adaxial and abaxial epidermis localized PIN1 directs auxin to the leaf primordium apex. PIN1 localized in inner cells then directs auxin downward, which induces procambial midvein specification [71,72]. In very early leaf primordia, PIN1 localization converges in the leaf margins, forming part of the middle domain [73]. Blocking PIN-mediated auxin transport between the SAM and leaf primordia via localized NPA or Brefeldin A treatment led to ectopic auxin signaling in the adaxial domain, inhibition of leaf margins, and the formation of axisymmetric leaves in tomato [61]. The same phenotype was also obtained via the ectopic application of auxin or an auxin analog to the adaxial domain [61], suggesting that PIN-mediated auxin transport is responsible for the adaxial low-auxin domain.

In fact, auxin transport might explain the Sussex signal, which is presumably produced in the SAM and transported to the adaxial domain to promote its formation. When an incipient primordium was separated from the SAM using a microsurgical incision, a radial axisymmetric leaf formed due to the blocking of the presumable Sussex signal [74]. Perhaps the microsurgical incision abolished epidermal auxin transport [61]. Indeed, auxin-induced *WOX* expression was significantly reduced after microsurgical incision, which is consistent with the axisymmetric leaf phenotype [21]. Although additional systematic analysis of PIN1 polarity is needed, it is likely that PIN-mediated auxin transport from the leaf primordium to the SAM serves as a signal, which is contrary to the original proposal that a SAM-derived signal conditions leaf flattening [61]. An alternative hypothesis is that wounding induces auxin depletion, which may activate *KAN1* in the adaxial domain and leaf abaxialization [43]. However, auxin maxima often overlap with *KAN1* expression in the shoot apex, questioning whether auxin suppresses *KAN1* expression.

It remains unknown how downstream effectors translate upstream signals into the determination of three-dimensional leaf shape. Auxin might also coordinate this shaping process. On the one hand, auxin promotes cell wall loosening [75,76,77]. Consistent with the transient adaxial low-auxin domain, the adaxial epidermis in tomato exhibits transient high mechanical elasticity compared to the abaxial epidermis [78]. The adaxial domain also exhibits transient high methyl esterification of cell wall pectins, which affects cell wall mechanics [79]. On the other hand, auxin-promoted *WOX* expression in the leaf margins of the middle domain is thought to increase the local growth rate. Computational modeling showed that the combination of differences in wall stiffness and increased leaf margin growth is sufficient to promote asymmetric shaping of the early primordium [80,81]. A microtubule-mediated mechanical feedback mechanism further amplifies the initial asymmetry to generate highly anisotropic blade growth [82].

Note that the amplification of asymmetry potentially provides a parsimonious explanation for leaf evolution, representing an alternative to the influential but highly questioned Zimmermann’s telome theory. Fossil evidence indicates that leaves evolved from lateral branches [83]. The telome theory proposes a series of shape transformations that have occurred over the course of evolution, but plausible molecular evidence is lacking [83,84]. According to the current theory, prepatterned gene expression in the shoot apex leads to an initial break in symmetry, which is in part mediated by auxin. Subsequently, the microtubule-mediated mechanical feedback mechanism amplifies the asymmetry to form a planar leaf blade [82].

## 4. The Regulation of Compound Leaf Patterning by Auxin

**Figure 3:**
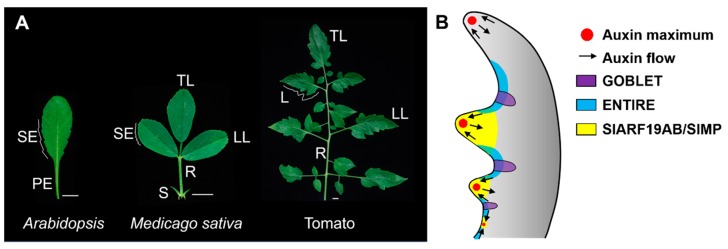
Morphology of simple and compound leaves. (**A**) Leaves of *Arabidopsis*, Alfalfa (*Medicago sativa*) and tomato. SE, serration; PE, petiole; TL, terminal leaflet; LL, lateral leaflet; R, rachis; S, stipule; L, lobe. Scale bars: 1 cm. (**B**) Model of the regulation of compound leaf development by auxin, *GOB*, *E*, and E-targeted *ARFs* in tomato.

Although leaf shape varies within and between plant species, leaves are either simple or compound based on the number of blades [85,86]. Simple leaves (in plants such as *Arabidopsis*) have a single blade attached to a petiole. Serrations may form along the margins of simple leaves. Compound leaves (in plants such as tomato and *Medicago truncatula*) have separate blades known as leaflets attached to a common rachis. Tomato leaves consist of a terminal leaflet and (usually) three pairs of lateral primary leaflets attached to a rachis (Figure 3). Each leaflet consists of a lamina supported by a petiolule. Some primary leaflets develop secondary leaflets. In addition, intercalary leaflets form between primary leaflets [86]. *Medicago* leaves consist of a terminal leaflet and two lateral leaflets attached to a rachis (Figure 3), with two stipules present at the leaf base [86]. Like the margins of simple leaves, leaflet margins can be smooth, serrated, or lobed. For example, tomato leaflets have lobed margins, and *Medicago* leaflets have serrated margins.

The highly diverse shapes of compound leaves rely on differences in leaf margin activity. Simple and compound leaves share similar developmental logic, but the differences between them determine shape variations. In particular, the balance between morphogenesis and differentiation is essential for determining leaf shape [87]. Morphogenesis is a transient indeterminate state in which cells actively divide and undergo patterning [88,89]. Differentiation is a gradual process that follows (and often overlaps with) morphogenesis. Cells stop undergoing division and begin to expand during differentiation [5]. This notion was supported by a cellular-resolution growth study comparing the simple leaf primordium of *Arabidopsis* with the compound leaf primordium of *Cardamine* (*Cardamine hirsute*), a close relative of *Arabidopsis*. In both species, the growth of margin circumference is anisotropic, whereas blade growth is isotropic [90]. Cell growth and proliferation are restricted to the leaf proximal region in *Arabidopsis* but are shifted to a more distal region in *Cardamine* leaves, resulting in delayed differentiation and prolonged morphogenesis and marginal patterning [90]. Both serrations and leaflets result from leaf margin patterning [91]. However, these two shapes are quantitatively different. Delayed differentiation and prolonged morphogenesis and patterning would convert serrations into leaflets [90].

Leaflet outgrowth is directed by auxin response maxima coinciding with PIN1 convergence points, a process highly similar to leaf initiation at the SAM [40,91,92,93]. Analysis of the expression of the marker DR5 in young tomato leaves showed that auxin signaling occurs at initiating leaflet positions but is repressed between leaflets [93,94]. An examination of the subcellular localization of PIN1 in tomato revealed that auxin is transported to leaflet initiation sites [93]. Inhibiting PIN-mediated auxin transport by NPA treatment converted compound leaves into simplified leaves [10,93]. Conversely, ectopic local auxin application induced ectopic leaf blade outgrowth or ectopic leaflet formation along the treated region [93,94]. Finally, ectopically expressing the auxin biosynthetic enzyme iaaM in early leaves also resulted in ectopic leaflet formation [94].

Many components of the auxin-signaling pathway significantly affect compound leaf formation or leaf serration (Figure 3). Aux/IAA proteins inhibit the auxin response by interacting with ARF activators to inhibit downstream gene expression [28]. Auxin induces Aux/IAA degradation by promoting the interaction between Aux/IAA and TRANSPORT INHIBITOR RESPONSE1/AUXIN SIGNALING F-BOX (TIR1/AFB) proteins, which belong to ubiquitin ligase complexes [28]. *ENTIRE* (*E*) is an Aux/IAA gene family member that is expressed in the intercalary regions between tomato leaflets [95,96]. Loss of function of *E* results in expanded DR5 signals throughout the entire leaf margin and ectopic blade growth in intercalary regions, leading to the formation of fused primary leaflets. *PIN1-GFP*, which is upregulated by auxin [31], is also upregulated in the intercalary regions of leaves [93,94]. Therefore, *E* inhibits auxin responses to maintain the bladeless identity of the rachis. *E* functions with its targeted ARFs to regulate tomato leaf shape [97,98].

E interacts with several ARF activators, including SIMP, SIARF19A, and SIARF19B, which have complementary expression patterns to E, i.e., expression in leaflet regions but absent or reduced expression in intercalary regions [98]. SIMP, SIARF19A, and SIARF19B quantitatively and unequally promote leaflet growth: *slarf* single mutants have reduced leaflet number, and *slarf* mutant combinations show enhanced and variable reduced leaflet phenotypes [98]. Multiple layers of ARF activators and suppressors function in the leaf margin to tune auxin activity and further stabilize leaf shape. Indeed, introducing *slarf* into the *e* mutant background partially rescued the *e* phenotype but with phenotypic variability, and introducing a mutation of the auxin transporter gene *slsopin1* into the *e slmp* double mutant led to the production of completely simplified leaves [98].

Several miR160-targeted ARFs in tomato that are putative suppressors also play a crucial role in compound leaf patterning. *SlmiR160* is expressed in provascular tissues, whereas miR160-targeted *SlARF10A* and *SlARF17* are expressed in leaflet regions. These miR160-targeted ARFs antagonize auxin responses in intercalary regions and promote leaflet separation. Indeed, plants overexpressing miR160-resistant forms of *SlARF10A*, *SlARF10B*, or *SlARF17* have increased leaf complexity and reduced lamina growth, and plants overexpressing *miR160* have reduced leaf complexity and ectopic lamina growth. Moreover, genetic evidence indicates that miR160-targeted *ARF*s and *E* act partially redundantly to locally inhibit lamina growth between leaflets [97].

The boundary-promoting NAC family genes also have important roles in compound leaf patterning. In tomato, the NAC gene *GOBLET* (*GOB*) promotes leaflet specification in tomato and is specifically expressed in narrow stripes between leaflets [99,100]. Loss-of-function *gob* mutants have simplified leaves [99]. Strikingly, a similar phenotype is found in *GOB* overexpression plants [99]. Spatiotemporal patterning of *GOB* activity alters the distribution of auxin signaling during leaflet patterning. In plants with reduced or enhanced *GOB* activity, auxin maxima are no longer regularly patterned. Furthermore, the simple leaf phenotype of *GOB*-overexpressing plants can be suppressed by inhibiting auxin transport and signaling. Combining *gob* and *e* mutations results in the complete abolishment of leaflet initiation, which is accompanied by the formation of uniform DR5 signals along leaf margins. These observations led to the proposal that *GOB* and *E* redundantly restrict auxin responses to promote compound leaf patterning [94] (Figure 3).

The NAC─auxin module is used during leaflet formation and the development of leaf serration [91,94,101]. In *Arabidopsis*, auxin response maxima (as detected using the DR5 reporter) precede the initiation of serration, whereas the *Arabidopsis GOB* ortholog *CUC2* expression was detected between serrations [91]. Disturbing the interspersed distribution of auxin maxima or *CUC2* expression results in leaves with smooth margins [91]. Experimental analysis and mathematical modeling showed that a feedback module between CUC2 and PIN-mediated auxin maxima is sufficient to explain the development of serration. In this feedback module, CUC2 promotes the establishment of PIN1 convergence points, thus promoting the establishment of auxin maxima, and auxin inhibits *CUC2* expression [91]. Independent studies in *Medicago* also support the roles of auxin transport and signaling in compound leaf patterning. *SMOOTH LEAF MARGIN1* (*SLM1*)/*MtPIN10* is the ortholog of *Arabidopsis PIN1* [102,103]. The loss-of-function *slm1* mutant has a reduced number of lateral leaflets and smooth leaflet margins [103]. As mentioned earlier, the NAC─auxin module functions in leaf boundary specification and organ separation, indicating that a regulatory module is often repetitively used in different developmental contents.

## 5. Conclusions

The leaf is a classical system in which to study organ growth and patterning and serves as the basis for many plant organs. Many recent studies have demonstrated that auxin plays crucial roles in various aspects of leaf development. In fact, auxin also regulates the development of vascular tissue, stomata, and trichomes [104,105,106], which are important leaf components. This is not surprising because PIN-mediated auxin transport forms a positive feedback loop to generate various patterns [107]. Nevertheless, recent high-resolution spatiotemporal analyses of auxin signaling distribution and PIN localization have pointed to the existence of previously unrecognized roles of auxin in leaf development. Live imaging at cellular resolution has also emerged as a pivotal tool to explore how auxin dynamically regulates leaf development.
